# PageRank as a method to rank biomedical literature by importance

**DOI:** 10.1186/s13029-015-0046-2

**Published:** 2015-12-09

**Authors:** Elliot J. Yates, Louise C. Dixon

**Affiliations:** College of Medical and Dental Sciences, University of Birmingham, Birmingham, B15 2TT UK

**Keywords:** PageRank, Bibliometrics, Citation count, Impact factor, Journal ranking

## Abstract

**Background:**

Optimal ranking of literature importance is vital in overcoming article overload. Existing ranking methods are typically based on raw citation counts, giving a sum of ‘inbound’ links with no consideration of citation importance. PageRank, an algorithm originally developed for ranking webpages at the search engine, Google, could potentially be adapted to bibliometrics to quantify the relative importance weightings of a citation network. This article seeks to validate such an approach on the freely available, PubMed Central open access subset (PMC-OAS) of biomedical literature.

**Results:**

On-demand cloud computing infrastructure was used to extract a citation network from over 600,000 full-text PMC-OAS articles. PageRanks and citation counts were calculated for each node in this network. PageRank is highly correlated with citation count (R = 0.905, *P* < 0.01) and we thus validate the former as a surrogate of literature importance. Furthermore, the algorithm can be run in trivial time on cheap, commodity cluster hardware, lowering the barrier of entry for resource-limited open access organisations.

**Conclusions:**

PageRank can be trivially computed on commodity cluster hardware and is linearly correlated with citation count. Given its putative benefits in quantifying relative importance, we suggest it may enrich the citation network, thereby overcoming the existing inadequacy of citation counts alone. We thus suggest PageRank as a feasible supplement to, or replacement of, existing bibliometric ranking methods.

## Background

MEDLINE is the premier bibliographic database of the U.S National Library of Medicine (NLM), containing over 22 million biomedicine-related entries. With approximately 750,000 new citations added in 2014, it is essential to identify literature of the highest quality for priority reading [1]. High citation rates (in addition to journal impact factor and circulation rates) are proposed to be predictive of article quality [2], thus in turn, scientific importance. Factors such as bias towards review articles and variable bibliographic lengths however suggest that such methods are not always optimal [3].

Citation counts give no weighting towards articles of greater importance. Naturally, definition of such importance is a subjective task. In a static system of inter-article referencing, we observe that a citation by an article from a low distribution journal has equivalence to a citation from a large-scale systematic review. Perhaps a weighting approach would favour articles of greater perceived ‘scientific gravity’, however this may neglect the emerging relevance of an article’s spread through the scientific community. Therefore a method of objectively weighting literature importance would be highly beneficial.

The PageRank algorithm, originally used for link analysis by the search engine, Google [4], provides one such method of ranking by importance. The concept, originally applied to web pages, proposes that a web page itself carries a greater importance if linked to by other high importance pages. Thus for a closed system of total web pages online, a system of merit can be constructed based on assigning a relative weighting (as a proportion of the entire database) to each web page.

Much as web pages are interconnected through hyperlinks, scientific articles are themselves linked via their citations. As such, this study seeks to investigate PageRank-based bibliometrics as an alternative to citation counts alone.

## Methods

The PubMed Central open access subset (PMC-OAS) represents a more liberally-licenced part of the PubMed Central collection [5], freely available online. Contributing journals provide selected full text articles in eXtensible Markup Language (XML) format, specifically for data mining purposes.

**Fig. 1:**
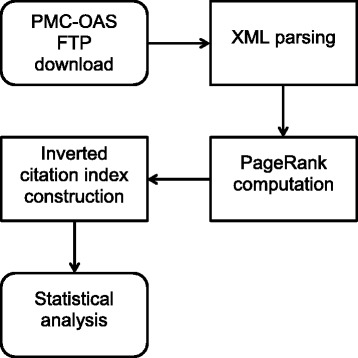
Methodology flowchart. Flowchart representing the major steps of data manipulation, as outlined in Methods

PMC-OAS was here chosen, both due to ease of accessibility, though also as a training corpus allowing concept validation prior to expansion to the entirety of MEDLINE. With over 600,000 unique manuscripts included, the dataset amounts to some 40Gb uncompressed [6]. Data parsing and computation was performed in three steps (Fig. 1).

### XML parsing

With data ingestion going beyond the capability of traditional desktop computing, on-demand cloud-computing infrastructure was leveraged to parallelise metadata extraction. This commodity cluster environment represents a readily-available, low-cost method of scaling up ‘embarrassingly parallel’ computational tasks [7].

XML parsing was performed in parallel on four compute nodes (2Gb RAM, 2 virtual CPU cores) using a hand-written Python [8] parser in under two hours (Appendix 1). PubMed identification (PMID) numbers of ‘outbound’ citations were extracted from each article’s reference list and used as reference keys for every citation vertex in the graph of article nodes.

### PageRank computation

**Fig. 2:**
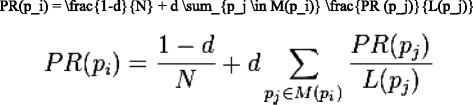
PageRank algorithm. PageRank algorithm representation. Set of unique PMIDs in citation network [*pi*], individual PageRank [*PR(pi)*], dampening factor [*d* = 0.85], total number of unique PMIDs [*N*], set of all inbound citations to *pi* [*M(pi)*], PageRank values of all inbound citations to *pi* [*PR(pj)*] and number of outbound citations of *pj* [*L(pj)*]

PageRank computation was performed on a single compute node (specifications as previous) using an open source C++ based implementation of the algorithm [9]. The algorithm can be summarised as per Fig. 2, where *pi* represents the set of all unique PMIDs in the citation network (and *PR(pi)* its individual PageRank), *d* is the dampening factor (d = 0.85 here), *N* is the total number of unique PMIDs, *M(pi)* represents the set of all inbound citations to *pi*, *PR(pj)* represents the PageRank values of all inbound citations to *pi* and *L(pj)* is the number of outbound citations of *pj*.

A dampening factor was originally introduced in PageRank to model an imaginary surfer randomly clicking on links, that will eventually stop clicking. 0.85 suggests an 85 % probability that at any step, this imaginary surfer will continue to click. Due to the recursive nature of the algorithm, a convergence value (epsilon) of 0.00001 was used to guarantee precision. The algorithm was used as per the reference implementation except where otherwise described.

### Inverted citation index creation

MapReduce, a programming model for large corpus processing, also developed at Google, was used to create an ‘inverted citation index’. This distributed computational approach allows near linear scalability with increasing cluster size [10], thus facilitating a route for future corpus expansion. The inverted citation index generates a list of ‘inbound’ citations for each article node in the graph, with a corresponding total citation count.

The high-level programming language, Pig [11] was used as a layer on top of MapReduce for near-natural language manipulation of the dataset. A Pig script was written to facilitate numeric comparison between derived citation count and calculated PageRank (Appendix 2).

### Statistical analysis

Statistical analysis was performed using IBM SPSS version 21.0.0.0 [12].

## Results

The PageRank algorithm processed and ranked a total of 6293819 unique PMIDs as graph nodes, with 24626354 vertices, representing corresponding outbound citations. A random, 5 % sample of the data was taken (using SPSS randomisation) for statistical analysis. This figure comfortably exceeds the sample size calculation (*n* = 385 required, Raosoft [13]), detailed in Appendix 3.

### PageRank is shown to be a surrogate of literature importance

**Fig. 3:**
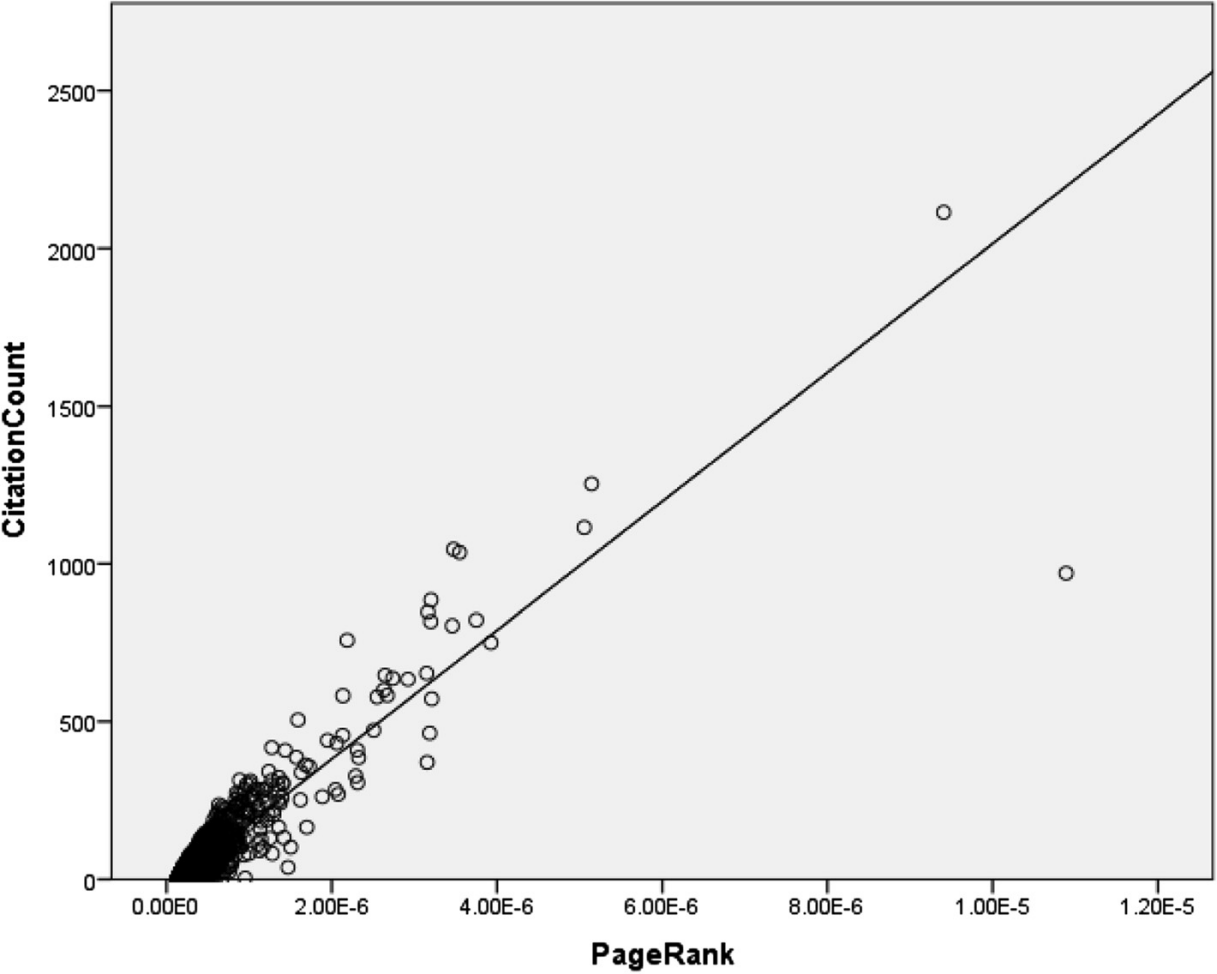
PageRank versus citation count. Scatter plot of PageRank versus citation count for random, 5 % sample of data. R = 0.905 (*P* < 0.01), R^2^ = 0.819 (*P* < 0.01)

A statistically significant correlation between PageRank and citation count was observed (*P* < 0.01) with a high correlation coefficient (R = 0.905). Simple linear regression was performed, obtaining R^2^ = 0.819 with the fitted regression line being statistically significant (*P* < 0.01), illustrated in Fig. 3.

As such, given the current role of citation count as a marker of literature importance, we demonstrate PageRank to be a similar such surrogate due to high degree of correlation. In light of this finding, we suggest that novel rankings would likely remain broadly similar and thus suggest that implementation of PageRank into the ranking of biomedical literature is feasible.

### Top of the corpus comparison

If the putative benefits of PageRank in quantifying importance are to be observed, it must be through outliers from those otherwise highly correlated with citation count. Such outliers may have been preferentially weighted by the algorithm, based on perceived importance. Due to the training subset size, it would be infeasible to account for such examples, however a top of corpus comparison allows some speculative inspection.

The top ten ranking articles of the corpus were compared by descending PageRank (Table 1). This table size was chosen for illustrational ease as graphical whole corpus analysis, aside from regression testing, was outside the scope of this research. From inspection, citation count decrement order matches that of PageRank (as expected from the high degree of correlation), with the exception of citation 11846609 (†), a method article with a lower relative PageRank ranking to its citation count.

**Table 1:** Top of the corpus comparison

PubMed ID (PMID)	Paper title	PageRank (E-5)	Citation count
9254694	Gapped BLAST and PSI-BLAST: a new generation of protein database search programs.	3.19	6291
2231712	Basic local alignment search tool.	2.88	5385
10802651	Gene ontology: tool for the unification of biology. The Gene Ontology Consortium.	2.37	4293
11846609	Analysis of relative gene expression data using real-time quantitative PCR and the 2(−Delta Delta C(T)) Method.	1.95	6012†
7984417	CLUSTAL W: improving the sensitivity of progressive multiple sequence alignment through sequence weighting, position-specific gap penalties and weight matrix choice.	1.78	3899
942051	A rapid and sensitive method for the quantitation of microgram quantities of protein utilizing the principle of protein-dye binding.	1.62	3850
21546353	MEGA5: molecular evolutionary genetics analysis using maximum likelihood, evolutionary distance, and maximum parsimony methods.	1.58	3431
17488738	MEGA4: Molecular Evolutionary Genetics Analysis (MEGA) software version 4.0.	1.47	3075
5432063	Cleavage of structural proteins during the assembly of the head of bacteriophage T4.	1.13	2881
3447015	The neighbor-joining method: a new method for reconstructing phylogenetic trees.	1.12	2171

Top of the corpus comparison (*n* = 10), sorted by PageRank, descending. Paper titles were sourced from PMID via PMC-OAS look-up, though were not included in the initial XML extraction. Rankings accurate as of January 2015

Whilst this represents a single example, we hypothesize that a method article is likely to be widely cited by those utilising its techniques, however this gives little information about the importance of such implementers. As such, we suggest that this correlation outlier has been proportionally ‘down-ranked’ by the PageRank algorithm in relation to the rest of the comparative head.

Whilst further work is required to validate such claims, we suggest this finding may build upon the notion of PageRank’s potential benefits in outweighing citation count alone. If the method is truly able to better weight those articles with higher importance rather than mass citation, we propose that its implementation into the ranking of biomedical literature may be warranted.

## Discussion

### PageRank can be trivially calculated on commodity cluster hardware

The use of on-demand cloud computing infrastructure for data extraction and computation allows for scalability with increasing corpus size. In the event of increasing article burden, additional XML parsing nodes could be employed with linear cost and throughput. Despite the uncompressed corpus totalling approximately 40Gb, the fully citation-extracted form was <500 Mb. Therefore, we suggest that growth by an order of magnitude (in the range of entire MEDLINE database size) could still be stored on a single commodity hard drive.

Whilst the PageRank calculation was performed on a single node, expansion beyond 2Gb of RAM on a single computer is becoming cheaper and widely available [14]. The use of MapReduce for inverted citation network creation allows near-linear scalability, similar to XML parsing, and can thus be trivially re-evaluated as the corpus grows. PMC-OAS is updated daily, thus all metrics can be recalculated in a matter of minutes (minus the cost of data parsing), as required by the maintainer.

### Expanding automated XML processing to MEDLINE as a whole is problematic

The PMC-OAS full-text articles are freely available in XML format, facilitating automated citation extraction. Unfortunately, the vast majority of MEDLINE articles are not open access, meaning that full-text access in not trivially available without bulk licencing programmes. Furthermore, the lack of XML-based metadata in non-open access articles limits the capability for rapid citation network generation.

Efforts have been made to parse bibliographic data from papers [15, 16], however attempts are limited by paid access to such articles in addition to the efficiency of extraction from a variety of article distribution file formats. We thus identify expansion beyond this 600,000-article training corpus as a major barrier to non-proprietary bibliometrics.

Articles appearing in PMC-OAS, referenced articles, which were not included in the corpus. This means that the latter’s PMID appeared in the citation network and thus received a PageRank. However, due to the limited inclusion set of this work, the PageRank (and thus relative ordering) is by no means final and would inevitably change should expansion to the whole of MEDLINE be feasible.

### Other methods of importance quantification

Thus far, importance analysis has been derived from article citation networks alone. However, importance is a non-static entity, with the impact of papers going beyond that of, who cites who. Indeed, importance of a particular work may be represented by its spread through the scientific community, rather than an ‘acknowledgement-based’ system of the traditional publishing model. Social media may provide a real-time window into this community dissemination.

Altmetrics, the use of the social web for insight into article impact [17], has previously shown promise in correlation with citation count and may therefore add to bibliometrics through real-time importance weighting [18]. Consideration of social impact is beyond the scope of this research, though provides an exciting avenue for further exploration, perhaps in conjunction with PageRank.

## Conclusions

PageRank is a novel method for determining the importance of biomedical literature. The possibility of commodity cluster hardware use and value re-calculation following corpus expansion suggests that curation of an open access citation network is not beyond the limits of a single maintainer. Whilst further work will inevitably be required to expand the network beyond the XML data-mining corpus of the PubMed Central open access subset, the 600,000-article training corpus provides a starting platform for PageRank’s addition to existing importance ranking methods.

## Appendix

### Appendix 1

Python source code for XML citation network extraction. Script finds .nxml files within the directory where all PMC-OAS XML full-text documents were extracted. Subsequently generates in key, value format corresponding [citer PMID, cited PMID] and stores as a comma separated value (CSV) file.

### Appendix 2

Pig source code for citation network generation and further PageRank comparison. Accepts key, value list of citer, cited PMIDs (as per Appendix 1) and groups by cited PMID before co-grouping with corresponding PageRank value and citation count.

### Appendix 3

Statistical analysis performed on derived dataset, accurate as of January 2015. Tables generated using IBM SPSS version 21.0.0.0.

Kolmogorov-Smirnov test for normality

**Table 2:** Tests of normality

	Kolmogorov-Smirnov^a^		
	Statistic	df	Sig.
PageRank	.383	314664	.000
CitationCount	.399	314664	.000

^a^Lilliefors Significance Correction

Pearson’s Correlation test

**Table 3:** Correlations

		PageRank	CitationCount
PageRank	Pearson Correlation	1	.905^a^
	Sig. (1-tailed)		.000
	N	314664	314664
CitationCount	Pearson Correlation	.905^a^	1
	Sig. (1-tailed)	.000	
	N	314664	314664

^a^Correlation is significant at the 0.01 level (1-tailed)

Simple Linear Regression

**Table 4:** Model summary

Model	R	R Square	Adjusted R Square	Std. Error of the Estimate
1	.905^a^	.819	.819	4.844

^a^Predictors: (Constant), PageRank

**Table 5:** ANOVA^a^

Model		Sum of Squares	df	Mean Square	F	Sig.
1	Regression	33389613.691	1	33389613.691	1423001.392	.000^b^
	Residual	7383297.502	314662	23.464		
	Total	40772911.193	314663			

^a^Dependent Variable: CitationCount

^b^Predictors: (Constant), PageRank

- •Equation for the regression line:
- ○ CitationCount = −27.861 + (204365203.423 x PageRank)
  **Table 6:** Coefficients^a^
  Model		Unstandardized Coefficients		Standardized Coefficients	t	Sig.
  		B	Std. Error	Beta		
  1	(Constant)	−27.861	.027		−1023.789	.000
  	PageRank	204365203.423	171318.510	.905	1192.896	.000
  ^a^Dependent Variable: CitationCount

## Abbreviations

PMC-OAS: PubMed Central open access subset
NLM: National Library of Medicine
XML: eXtensible Markup Language
PMID: PubMed identification
FTP: File Transfer Protocol
CSV: Comma-separated values
